# Sexual dimorphism in the hepatic protein response to a moderate trans fat diet in senescence-accelerated mice

**DOI:** 10.1186/s12944-017-0639-7

**Published:** 2017-12-13

**Authors:** Steven A. Bloomer, Kathryn E. Wellen, Gregory C. Henderson

**Affiliations:** 1 Division of Science and Engineering, Penn State University, Abington College, 1600 Woodland Rd, Abington, PA 19001 USA; 2 Department of Cancer Biology, University of Pennsylvania Perelman School of Medicine, Philadelphia, PA 19104 USA; 3 Abramson Family Cancer Research Institute, University of Pennsylvania Perelman School of Medicine, Philadelphia, PA 19104 USA; 4 Rutgers University, New Brunswick, NJ USA

**Keywords:** ATP citrate lyase, Fatty acid synthase, MCP-1, Inflammation, Oxidative stress

## Abstract

**Background:**

Aging is characterized by increases in inflammation and oxidative stress, conditions that are exacerbated by environmental factors such as diet. In this study, we investigated the effects of a *trans*-fatty acid (TFA) diet on the liver in adult (25 wk) and old (60 wk) senescence-accelerated mice (SAMP8 strain) of both sexes. Our goal was to assess the effects of the diet on protein markers of inflammation and oxidative stress in the liver.

**Methods:**

Male and female mice were placed on life-long diets containing similar amounts of total fat (17%), with differing amounts of TFA: 2% (moderate TFA group) or 0.2% of total energy from TFA (control diet group). At the indicated ages, livers were harvested and evaluated for markers of inflammation and oxidative stress, as well as for enzymes of fat metabolism via immunoblotting. Relative densities of protein bands were determined and compared via a three-factor ANOVA.

**Results:**

Compared to males, females demonstrated significantly lower inflammatory protein expression (ICAM-1, MCP-1, COX-2), along with lower expression of the DNA damage marker, Gadd153, and the oxidative stress marker, HO-1. Female mice demonstrated higher expression of antioxidant enzymes (SOD-1, SOD-2, and Ref-1) and lipogenic enzymes (FASN, ACLY) compared to male mice. While HO-1 was elevated in the female mice fed the TFA diet compared to controls, the diet did not affect other markers of oxidative stress or inflammation. However, the diet was associated with significant increases in FASN and ACLY in adult (25 wk) male mice.

**Conclusions:**

Our results suggest sexually dimorphic protein expression in the liver, with female mice demonstrating lower inflammation and increased oxidative stress defenses. Additionally, considering that FASN and ACLY contribute to hepatic lipogenesis, our results suggest a potential mechanism for the dyslipidemia in adult male mice that is associated with TFA diets.

## Background

While aging per se is associated with exaggerated inflammation and oxidative stress systemically, physiological perturbations can augment these changes, and lead to a more rapid decline in physiological function. The liver is an example of an organ that undergoes these age-related changes [1, 2], and it is also exquisitely sensitive to changes in environmental conditions, particularly diet. For example, many investigations have evaluated the effects of a high fat diet on liver homeostasis in young organisms, demonstrating that these diets are associated with elevations in inflammation and oxidative stress [3–5]. However, less attention has focused on the effects of a dietary intervention on the liver in a model of aging. A recent study examined the effects of a short-term (9-week) high-fat diet in C57BL/6 J mice aged 8, 16, and 52 weeks, and demonstrated that this diet increased the production of superoxide from hepatic mitochondria in each age group [6]. In another study, aged mice (20-month-old) fed a high-fat diet for 4 months demonstrated increased inflammation, oxidative stress, and fibrosis in the liver, compared to aged mice on the control diet [7]. Therefore, dietary manipulations can exacerbate conditions already associated with aging (i.e. augmented oxidative stress and damage). Since the number of individuals aged 80 and older in the world increases steadily, and the risk for liver pathology increases with aging [8], determining the effects of dietary manipulations on the potential for age-related pathologies is an important area of investigation.

Both the quantity and type of fat are important in mediating pathophysiological effects. Specifically, diets high in *trans*-fatty acids (TFA) from partially hydrogenated oils are particularly harmful because they increase the risk for coronary heart disease and metabolic syndrome [9]. Furthermore, diets high in TFA are associated with an increase in oxidative stress in the liver [5]. While much research has correlated TFA intake with morbidity in humans, few studies have directly modified dietary TFA in an aging model. The studies described earlier on aging and high fat diets did not manipulate TFA, and were short-term in nature [6, 7]. Therefore, the effects of long-term, moderate TFA intake on inflammation and oxidative stress in the liver with aging are unclear. Our previous work demonstrated that life-long intake of 2% of total energy from TFA (an intake typical of the American diet [9]) in senescence-accelerated mice (SAMP8 strain) was associated with increases in plasma triglyceride and total cholesterol, suggesting an effect of TFA on the liver – an organ that regulates plasma fat and cholesterol [10]. Furthermore, we observed that TFA exacerbated the age-related decline in skeletal muscle strength in both sexes of mice [10]. Thus, even a moderate amount of TFA can result in dyslipidemia and organ dysfunction with aging.

In the current investigation, our goal was to evaluate further the effects of this moderate TFA diet and aging on the liver in both sexes of the SAMP8 mouse strain. These mice are useful for studying the aging process because they are prone to age-related changes such as increases in hepatic inflammation and oxidative stress [11, 12]. Considering that TFA intake is associated with similar changes [5, 13], we hypothesized that the TFA diet would exacerbate age-related liver injury in aged SAMP8 mice. To assess liver phenotype in this model, we evaluated well-accepted protein markers of inflammation and oxidative stress. In this report, we demonstrate that female sex was associated with lower expression of inflammatory markers and higher expression of antioxidant, as well as lipogenic enzymes. Importantly, we also demonstrate that the lipogenic enzymes, fatty acid synthase (FASN) and ATP citrate lyase (ACLY) increased in adult male mice fed the trans fat diet, which could explain the increased plasma triglycerides and cholesterol observed previously in this group [10].

## Methods

### Animal experiments

All animal protocols were approved by the Rutgers University Animal Care and Facilities Committee (protocol 10–011), and performed in accordance with the National Institutes of Health guide for the care and use of laboratory animals. All mice were given food and water ad-libitum, and were maintained in a temperature-controlled room on a 12–12 light-dark cycle. Each sex of Senescence Accelerated Mice Prone-8 (SAMP8; Harlan Laboratories- Indianapolis, IN) were randomly divided into four groups: adult (25 weeks old) control diet; adult *trans*-fat (TFA) diet; old (60 weeks old) control diet; and old TFA diet, resulting in 8 total groups with 5–7 animals in each group. Animals were placed on each diet at 3 weeks of age and remained on the diets until euthanization. The macronutrient content of both diets was 63% carbohydrate, 20% protein, and 17% fat. The TFA diet consisted of 2% of total energy from TFA, from vegetable shortening (partially hydrogenated vegetable oil). The control diet contained 0.2% of total energy from TFA and did not contain vegetable shortening. The dietary fatty acid profile of each diet has been described in detail previously [10].

### Tissue collection and processing

At 25 (adult) or 60 (old) weeks of age, mice were anesthetized with a lethal dose of pentobarbital sodium (100 mg/kg). Livers, quadriceps, and plasma were quickly removed and frozen in liquid nitrogen. The analysis of skeletal muscle and blood parameters has been reported previously [10]. Frozen livers were ground under liquid nitrogen and homogenized with lysis buffer (50 mM Tris pH 7.4, 150 mM NaCl, 0.25% sodium deoxycholate, 1% Triton-X, 1 mM EDTA, 1 mM sodium vanadate) with HALT protease inhibitor cocktail (Thermo Fisher 78,430). The protein concentration of each sample was analyzed using the Bradford assay (BioRad, Hercules, CA), and equal amounts of proteins were added to sample buffer, and subsequently frozen until immunoblot analysis. All samples (except when probing for 4HNE) were boiled for 5 min before being loaded.

### Immunoblotting

Equal amounts of protein (50 μg) from whole liver lysates were separated on 12% polyacrylamide gels and transferred to nitrocellulose membranes. Membranes were blocked in 5% milk in tris-buffered saline with tween (TBST) for 30 min at room temp, and then incubated in primary antibody at 4 °C overnight. The primary antibodies and concentrations utilized are given in Table 1.

**Table 1:** Antibodies and dilutions

Antigen	Company	Catalog number	Dilution
ICAM-1	R&D Systems	AF583	1:250
MCP-1	Abcam	ab-25,124	1:1000
COX-2	Cell Signaling	12,282	1:250
Ref-1	Santa Cruz Biotechnologies	sc-17,774	1:1000
γ-GCS	Santa Cruz Biotechnologies	sc-22,755	1:2000
SOD-1	Enzo Life Sciences	ADI-SOD-100	1:5000
SOD-2	Enzo Life Sciences	ADI-SOD-110	1:5000
Gadd-153	Santa Cruz Biotechnologies	sc-575	1:500
HO-1	Enzo Life Sciences	ADI-OSA-111	1:1000
FASN	Cell Signaling	3189	1:1000
ACLY	From ref. [54]		1:1000
4-hydroxynonenal	Abcam	46,545	1:500

After incubation in primary antibody, membranes were washed with TBST, and then incubated in secondary antibody (anti-mouse HRP 1:4000; Amersham NA931V or anti-rabbit HRP, 1:4000; Santa Cruz Biotechnologies # 2030). After incubation in secondary antibody, membranes were washed, and then treated with chemiluminescent substrate (SuperSignal® West Pico, Thermo Scientific). Images were developed using the Chemi-Doc XRS system (BioRad), and the brightness of bands was quantified using the Image lab program (BioRad). To ensure equal loading and transfer, membranes were then stained with Ponceau-S staining solution. The intensity of staining was quantified with Image lab, and the band density of the protein of interest was normalized to the intensity of the Ponceau stain. We and others have shown that protein abundance on nitrocellulose membranes is better quantified with Ponceau staining compared to routine protein loading controls such as beta-actin [14, 15]. Results were further normalized to the adult male control diet group, which was given a value of 1.

### Statistics

A 3-factor ANOVA (factors: age-sex-diet) was utilized to determine significant differences among groups. When first indicated significant (*p*-value less than 0.05) by the ANOVA, T-tests were subsequently performed to determine differences between groups using a Bonferroni *p*-value adjustment (0.05/number of tests) to correct for multiple comparisons.

## Results

### Inflammatory markers

**Fig. 1:**
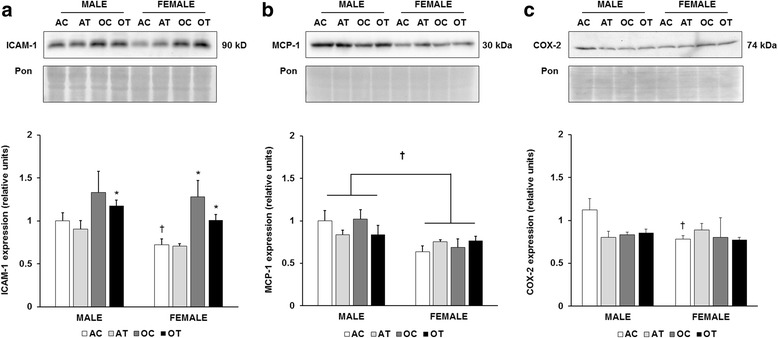
Decreased abundance of inflammatory proteins in female mice. Quantitation of ICAM-1 (**a**), MCP-1 (**b**), and COX-2 (**c**) protein abundance in the liver, normalized to the Ponceau stain, and further normalized to the adult, male control group. Top panels: representative immunoblots for target protein expression and Ponceau-stained membranes. Bottom panels: quantitation of target protein expression. Results are expressed as means + S.E.M., *n* = 5–7 animals per group. † Significant effect of sex within an age or diet group. Brackets designate a significant main effect of sex. * Significant effect of aging within a diet and sex group. AC: adult control diet; AT: adult *trans*-fat diet; OC: old control diet; OT: old *trans*-fat diet

Since both aging and TFA diets are associated with a pro-inflammatory environment, we evaluated the expression of intercellular adhesion molecule-1 (ICAM-1), monocyte chemoattractant protein-1 (MCP-1), and cyclooxygenase-2 (COX-2). In male mice fed the control diet, ICAM-1 tended to increase with aging, but this difference did not reach statistical significance, likely due to the variability in the old group. However, in male mice fed the TFA diet, ICAM-1 was significantly elevated with aging (Fig. 1a). In the adult mice fed the control diet, ICAM-1 expression was significantly lower in the female mice compared to the male mice (*p* = 0.02). In the TFA-fed groups, the difference approached significance (*p* = 0.053). In the female mice, aging increased ICAM-1 expression in both the control (*p* = 0.019) and TFA diet groups (*p* = 0.003; Fig. 1a). However, the expression of ICAM-1 was not affected by the TFA diet in any of the groups. A significant main effect of sex was observed in MCP-1 expression (*p* = 0.002), with significantly lower protein abundance in the female mice (Fig. 1b). However, there were no significant effects of aging or diet on MCP-1 expression. In the adult mice fed the control diet, female mice demonstrated lower COX-2 protein expression compared to male mice (Fig. 1c). Like MCP-1, there were no significant effects of age or diet on COX-2 expression.

### Antioxidant enzymes and markers of oxidative stress

**Fig. 2:**
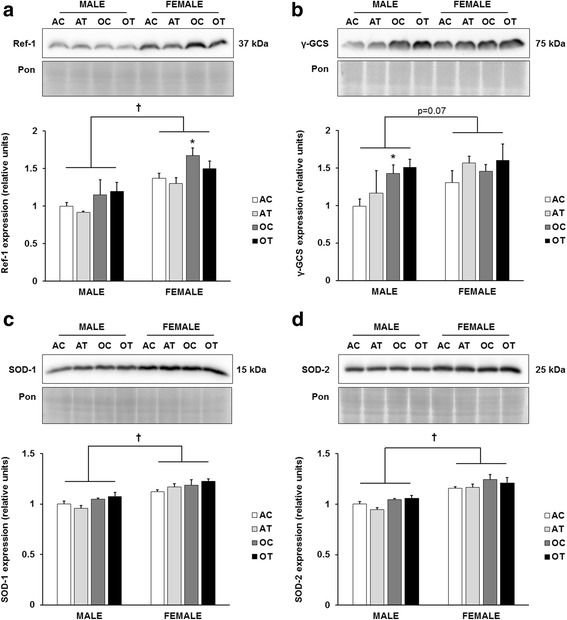
Female sex increases antioxidant protein expression. Quantitation of Ref-1 (**a**), γ-GCS (**b**), SOD-1 (**c**), and SOD-2 (**d**) protein abundance in the liver, normalized to the Ponceau stain, and further normalized to the adult, male control group. Top panels: representative immunoblots for target protein expression and Ponceau-stained membranes. Bottom panels: quantitation of target protein expression. Results are expressed as means + S.E.M., *n* = 5–7 animals per group. † Significant main effect of sex. * Significant effect of aging within a diet and sex group. AC: adult control diet; AT: adult *trans*-fat diet; OC: old control diet; OT: old *trans*-fat diet

To evaluate the redox environment of the liver, we determined the expression of several antioxidant enzymes involved in oxidative stress defense. We observed a significant main effect of age (*p* = 0.002) on redox factor-1 (Ref-1), which reached significance in the female mice on the control diet (Fig. 2a). Additionally, we observed a significant main effect of sex (*p* < 0.001) on Ref-1, with overall higher expression in female mice. There was a significant effect of age on protein levels of gamma glutamyl cysteine synthetase (γ-GCS), which reached significance only in the male mice on the control diet (Fig. 2b). Similar to Ref-1, there was a trend for γ-GCS to be higher in female mice (*p* = 0.07). We also evaluated two isoforms of superoxide dismutase (SOD): the cytosolic copper-zinc SOD (SOD-1; Fig. 2c), and the mitochondrial manganese SOD (SOD-2; Fig. 2d). Protein abundance of SOD-1 and SOD-2 in female mice was greater than in males (*p* < 0.001), but there were no significant effects of age or diet on either isoform. To evaluate oxidative damage in this model, we analyzed proteins modified with the lipid peroxidation adduct, 4-hydroxynonenal (4-HNE), which accumulates with aging and liver injury [16, 17]. The abundance of 4-HNE-modified proteins did not change with age, sex, or diet in this model (data not shown).

**Fig. 3:**
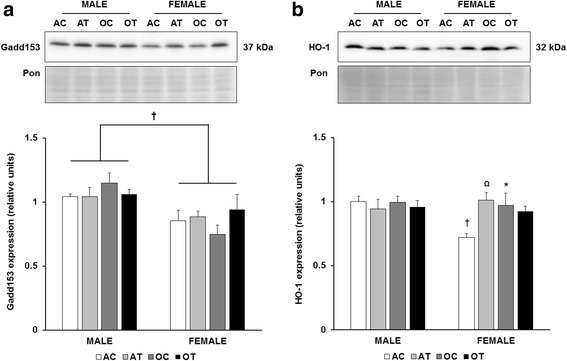
Decreased expression of Gadd153 and HO-1 in female mice. Quantitation of Gadd153 (**a**), and HO-1 (**b**) protein abundance in the liver. Top panels: representative immunoblots for target protein expression and Ponceau-stained membranes. Bottom panels: quantitation of target protein expression. Results are expressed as means + S.E.M., *n* = 5–7 animals per group. † Significant effect of sex within an age or diet group Brackets designate a significant main effect of sex. Ω Significant effect of *trans*-fat diet within an age and sex group. * Significant effect of age within a sex and diet group. AC: adult control diet; AT: adult *trans*-fat diet; OC: old control diet; OT: old *trans*-fat diet

To further probe the redox environment, we evaluated the expression of growth arrest and DNA damage-inducible protein 153 (Gadd153), and heme oxygenase-1 (HO-1). Female mice demonstrated overall lower expression of Gadd153 (*p* = 0.003), but there were no significant effects of aging or diet (Fig. 3a). Compared to adult male mice, HO-1 was significantly lower in adult female mice on the control diet (Fig. 3b). Both aging and the TFA diet increased HO-1 expression in female mice (Fig. 3b). Male mice did not exhibit differences in HO-1 expression with aging or diet.

### Lipogenic enzymes

**Fig. 4:**
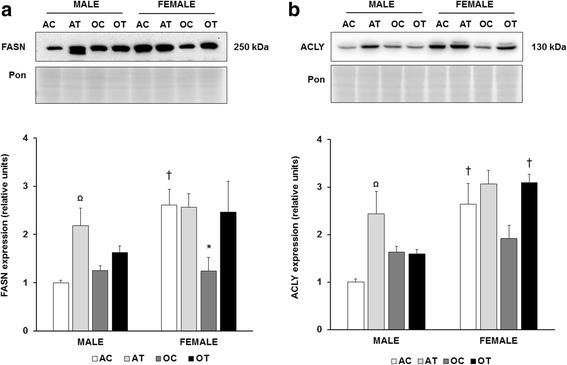
Effects of diet, age, and sex on lipogenic enzyme expression. Quantitation of fatty acid synthase (FASN; **a**) and ATP citrate lyase (ACLY; **b**) abundance in the liver. Top panels: representative immunoblots for target protein expression and Ponceau-stained membranes. Bottom panels: quantitation of target protein expression. Results are expressed as means + S.E.M., *n* = 5–7 animals per group. Ω Significant effect of *trans*-fat diet. † Significant effect of sex. *Significant effect of age within a sex and diet group. AC: adult control diet; AT: adult *trans*-fat diet; OC: old control diet; OT: old *trans*-fat diet

Finally, to verify an effect of dietary fat manipulation on enzymes involved in hepatic lipid synthesis, we investigated fatty acid synthase (FASN) and ATP citrate lyase (ACLY). Significant main effects of diet, age, and sex were determined for FASN expression (*p* = 0.004 diet; *p* = 0.001 sex; *p* = 0.044 age, Fig. 4a). In adult male mice, FASN expression increased approximately 2-fold with the TFA diet. In adult control mice, FASN was approximately 2.5-fold higher in female, compared to male mice. There was no effect of TFA on FASN in adult female mice. However, we observed a significant decrease in FASN expression in aged compared to adult female mice (*p* = 0.01). Also, we observed significant main effects of sex (*p* < 0.001) and diet (p < 0.001) on ACLY expression (Fig. 4b). Similar to FASN, adult female mice demonstrated a 2.5-fold elevation in ACLY expression, compared to adult male mice. The TFA diet increased ACLY in male, but not in female mice. There was no significant effect of age on hepatic ACLY protein.

## Discussion

In this report, we have shown novel effects of sex, age, and TFA on hepatic homeostasis in SAMP8 mice. Our investigation utilized a moderate level of TFA (typical of an American diet [9]) that was given for the entire life span of the mice; thus our study is especially relevant to long-term, or even life-long diets in humans. The major observations of this study were sexual dimorphism in expression of lipogenic and antioxidant proteins (higher in female than in male mice), and TFA-induced increases in lipogenic enzymes in adult male mice.

Interestingly, we did not observe significant effects of aging on several of the markers of oxidative stress or inflammation that were assessed in this study. Because the animals utilized are a model of accelerated aging, it is possible that these markers had already reached a plateau at 25 weeks of age (~6 months), and did not change further at 60 weeks of age (14 months). The results of recent studies support this concept. For example, hepatic lipid peroxidation is lower in 3- compared to 6-month-old SAMP8 mice, while values in 6- and 9-month-old mice are similar [18]. Also, decreased hepatic oxidative damage was observed in 1-month, compared to 6-month-old SAMP8 mice [19]. Finally, 2-month-old SAMP8 mice demonstrate lower inflammation compared to 10-month old SAMP8 mice [12]. Hence, there is considerable variability in the ages of SAMP8 mice utilized for aging studies, and very chronologically young SAMP8 mice tend to demonstrate lower inflammation and oxidative injury in the liver. We chose to use mice at 25 weeks of age to obviate the inclusion of juvenile or adolescent animals, still in stages of active growth and development [20]. Furthermore, SAMP8 mice at 25 weeks demonstrate 100% survival, and do not suffer from age-related conditions such as sarcopenia [21]. In future studies, it will be important to perform a more comprehensive evaluation of inflammation and oxidative stress in multiple organs using several age groups.

Contrary to our expectations, the TFA diet utilized in the present study did not increase the expression of ICAM-1, MCP-1 or COX-2, which are commonly used protein markers to assess the proinflammatory environment [22–24]. A previous study that did observe an increase in inflammation utilized a very high TFA diet – 57.3% of dietary fatty acids in the form of trans fats [13], compared to 11.82% in our study. However, consistent with our observations of a benign inflammatory effect of TFA on the liver, the authors of that study did not observe an effect of TFA on hepatic necrosis [13]. Thus, it appears that greater amounts of TFA in the diet, specifically intake levels beyond those typically consumed, are required to elicit hepatic inflammation.

Paralleling the lack of inflammatory protein induction, with the exception of HO-1 in female mice, the TFA diet also did not elicit increases in antioxidant proteins, or in 4-HNE-modified proteins. The induction of HO-1 in response to TFA is consistent with its role as a stress-responsive and/or redox-sensitive protein marker that is responsive to dietary manipulations [25]. The reasons for the lack of change in the other antioxidant proteins is not entirely clear, but this result suggests a specific effect of TFA on HO-1 expression. A generalized response to oxidative stress includes the upregulation of Ref-1, γ-GCS, and the SOD enzymes [26–28]; thus our results suggest that the amount of TFA utilized in our model did not substantially alter the redox environment of the liver. Our previous finding of a lack of a change in protein carbonyls in skeletal muscle [10] supports our current results in the liver.

The most striking findings in this investigation may be the sex-related differences in hepatic protein expression. For example, female mice displayed higher expression of antioxidant proteins, and lower expression of the inflammatory markers, ICAM-1, MCP-1, and COX-2. Furthermore, female mice demonstrated lower expression of Gadd153 and HO-1. Since Gadd153 protein is induced after DNA damage [29], and HO-1 protein is induced by oxidative stress [30], these results suggest that female sex is associated with lower DNA damage and a decreased oxidative burden within the cell. While it could be argued that decreased HO-1 abundance in female mice could reflect lower defenses against oxidative stress, HO-1 can also have a prooxidative role [31]; therefore, in this model, the patterns of change in the expression of HO-1 are consistent with its role as a sensor of oxidative stress. The higher expression of the SOD isoforms in female mice is consistent with a previous investigation [32]. Likewise, the higher levels of Ref-1 (a DNA repair enzyme) and lower levels of Gadd153 in female mice are consistent with the lower DNA damage observed in the livers of female rats [32]. To our knowledge, sex-related changes in Ref-1 and γ-GCS with aging have not been investigated in SAMP8 mice. Overall, this maintenance of antioxidant protein expression and the lower proinflammatory protein expression in female mice support the general finding of increased longevity in females, and is consistent with both the “Free Radical Theory of Aging” and the theory of “Inflammaging.”

Similar to the redox-sensitive proteins, FASN and ACLY were significantly elevated in female, compared to male mice, which suggests that estrogens (or other aspects of sexually dimorphic hormone profiles) exert a stimulatory effect on the expression of these proteins. Indeed, results in cell culture and in vivo support that estradiol increases FASN expression and activity [33, 34]. Since aged female mice experience a decrease in estradiol [35], our observation of a significant decrease in FASN expression in this group further supports a stimulatory role of estrogen. There is also evidence for a stimulatory role of estrogen in the expression of ACLY [36]. Overall, the similar trends in ACLY and FASN protein levels in our model suggest that estradiol influences the expression of both enzymes.

In addition to the significant effect of female sex on ACLY and FASN, these enzymes were increased in adult male mice on the TFA diet. These results are consistent with the findings of Cassagno et al. who demonstrated an approximate 3-fold increase in FASN mRNA in mice fed a 5% TFA diet [37]. On high-fat diets, FASN and ACLY expression are suppressed [38–40]. Since high-fat diets tend to be low in carbohydrates, and carbohydrates stimulate lipogenic gene expression [41], it is possible that the low carbohydrate content resulted in lower FASN and ACLY expression in those diets. It should be noted that in the present study, the total carbohydrate and fat content was similar between control and TFA diets (i.e. both diets contained 63% carbohydrate and 17% fat). Therefore, our observations demonstrate a response specific to altering the dietary proportion of TFA. The increase in these hepatic enzymes in the adult male TFA group was also associated with significantly increased plasma triglyceride [10], suggesting that the effect of TFA in increasing triglycerides is mediated by alterations in the liver. The exact mechanism through which TFA stimulates expression of these enzymes deserves further investigation, especially considering that de novo lipogenesis in the liver contributes to hepatic steatosis and metabolic syndrome [42], and that TFA intake in humans increases the risk for these diseases [9].

In light of this study’s observations, it is important to discuss sex differences in the susceptibility to metabolic diseases. Specifically, non-alcoholic fatty liver disease (NAFLD) is associated with lipogenesis, inflammation, and oxidative stress, and it is a sexually dimorphic disease with generally higher prevalence in males [43–46]. The involvement of sexually dimorphic hormone profiles in the pathogenesis of the disease is further underscored by the observation that after menopause, the incidence of NAFLD in males and females is approximately equal [46]. The male mice in our study demonstrated greater inflammation and oxidative stress, both of which are implicated in the development of steatohepatitis in rodent models [25, 47, 48]. Since significant correlations exist between markers of oxidative injury and inflammation in humans with NAFLD [49, 50], these rodent models accurately recapitulate several features of the human disease. Our previous results demonstrated that the TFA-induced increases in plasma triglycerides and cholesterol were greater in males than in females [10]. Thus, combined, our results suggest that on the background of lower oxidative stress and inflammation as observed in female mice, the effects of TFA on the liver are lessened, which would lower the susceptibility of female mice to liver disease. However, it is important to note that our results do not separate between genetic and gonadal sex; therefore, future studies on the effects of TFA distinguishing between chromosomal genotype and sex-related endocrine phenotypes may be useful [51, 52]. Nevertheless, the results in this paper suggest that sexual dimorphism in hepatic homeostasis might influence the predisposition to hepatic diseases.

### Limitations

It is important to consider the limitations of this report, and to identify analyses that could be useful in future studies on this topic of investigation. First, we did not evaluate these markers at the mRNA level, which could provide insights into potential sexual dimorphism in transcriptional regulation of these genes. In future studies, it will be important to perform mechanistic studies analyzing the effects of sex hormones on the regulation of specific transcripts such as FASN or ICAM-1. Second, during the organ harvest, the samples were not prepared for histology, precluding us from visualizing fat deposits in the liver, which would further confirm the TFA-induced lipid dysregulation. Finally, it is possible that a more thorough histological evaluation could reveal potential inflammatory effects of the TFA diet. However, given the lack of change in inflammatory markers and plasma TNFα [10] in the mice fed the TFA diet, we believe that robust histological inflammation would be unlikely in this model. It is also possible that inflammatory changes evident at the protein level could exist without overt histopathological changes [53].

## Conclusions

Taken together, the results of our current and previous [10] investigations suggest that even a modest increase in TFA intake can result in lipid dysregulation in adult male mice, which appears to be mediated by the effects of TFA on the liver. Since our results parallel findings in humans who consume high TFA diets, the complete removal of TFA from the diet should be considered to prevent the development of metabolic diseases.

## Abbreviations

4-HNE: 4-hydroxynonenal
ACLY: ATP citrate lyase
COX-2: Cyclooxygenase 2
EDTA: Ethylenediaminetetraacetic acid
FASN: Fatty acid synthase
Gadd153: Growth arrest and DNA damage-inducible protein 153
HO-1: Heme oxygenase 1
HRP: Horse radish peroxidase
ICAM-1: Intercellular adhesion molecule 1
MCP-1: Monocyte chemoattractant protein-1
NAFLD: Non-alcoholic fatty liver disease
Ref-1: Redox factor-1
SAMP8: Senescence accelerated prone mouse strain 8
SOD-1: Superoxide dismutase 1
SOD-2: Superoxide dismutase 2
TBST: Tris-buffered saline with tween
TFA: Trans-fatty acids
γ-GCS: gamma glutamyl cysteine synthetase
